# Immunosuppression Facilitates the Reactivation of Latent Papillomavirus Infections

**DOI:** 10.1128/JVI.02589-13

**Published:** 2014-01

**Authors:** G. A. Maglennon, P. B. McIntosh, J. Doorbar

**Affiliations:** a National Institute for Medical Research, Mill Hill, London, United Kingdom; b Royal Veterinary College, North Mymms, Hatfield, United Kingdom; c Department of Pathology, University of Cambridge, Cambridge, United Kingdom

## Abstract

At mucosal sites, papillomavirus genomes can persist in the epithelial basal layer following immune-mediated regression. Subsequent T-cell depletion stimulates a 3- to 5-log increase in the viral copy number, to levels associated with productive infection. Reappearance of microlesions was rare within the short time frame of our experiments but was observed in one instance. Our studies provide direct evidence that immunosuppression can trigger the reactivation of latent papillomavirus genomes, as previously proposed in humans.

## TEXT

Definitive evidence of human papillomavirus (HPV) latency has been difficult to obtain, and for the most part has been limited to anecdotal reports of recurrence in individuals. The prevalence of HPV disease in immunosuppressed patients is thought to result from the reactivation of latent infections (1–8), and as reviewed by Gravitt, there is growing evidence that HPV latency and the persistence of particular HPV types (even in the absence of apparent disease) constitutes an important risk factor in the development of cervical neoplasia (9, 10). The ability of papillomaviruses to persist long-term in a latent state (i.e., in the absence of disease) has been shown most convincingly in animal models and has led to the suggestion that latency may be a general consequence of immune-mediated regression (11–14). The low-titer inoculation of cottontail rabbit papillomavirus (CRPV) can lead to an asymptomatic cutaneous infection that can be activated by UV light to form lesions (14), and more recently, we have shown that rabbit oral papillomavirus (ROPV) can persist for a year or more in infected oral mucosa following immune-mediated regression of productive infection (12). In the ROPV study of immune-mediated latency, viral genomes were found by laser capture microscopy to be restricted to the epithelial basal layer at the site of previous infection, without significant genome amplification or virion production in the differentiated cell layers (12). In humans, the suppression of T-cell immunity, either by the administration of drugs such as cyclosporine to renal transplant recipients or following human immunodeficiency virus (HIV) infection, has been proposed as a cause of latent HPV reactivation at sites of previous disease (1–8). Waning T-cell immunity may similarly explain the increased detection of HPV in older women who lack signs of cervical disease by cytology. Because of the difficulties of demonstrating papillomavirus reactivation in humans, we decided instead to assess whether suppression of T-cell immunity in latently ROPV-infected rabbits (i.e., where the site of previous infection is known) might provide clearer evidence of reactivation following immune-mediated regression and allow us to establish whether immune changes can regulate the basal cell copy number and the ability to detect infection, as has been suggested in humans.

### Immunosuppression prevents spontaneous ROPV papilloma regression.

**FIG 1:**
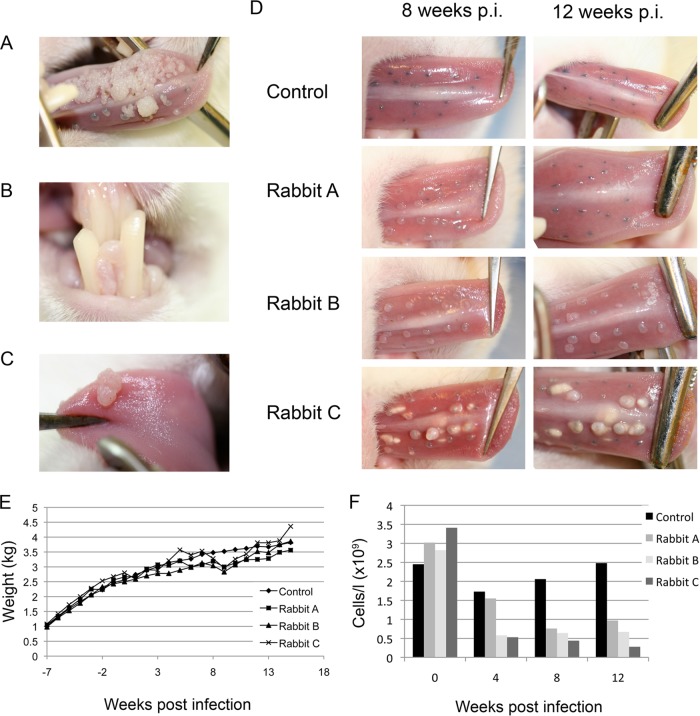
(A) Presence of florid ROPV-induced warts in immunosuppressed rabbits at 15 weeks postinfection. In immunocompetent rabbits, lesions were not detectable beyond 10 weeks. (B and C) In the immunosuppressed rabbits, secondary infections were also apparent at nonscarified sites by 15 weeks postinfection (p.i.). (D) Lesion size and abundance varied among immunosuppressed rabbits (rabbits A, B, and C), depending on the extent of immunosuppression (see panel F). Immunocompetent rabbits developed much smaller lesions (control), with tattoo marks indicating the sites of previous infection by week 12. (E) The cyclosporine-dexamethasone immunosuppression regimen was found not to have a major effect on weight gain in juvenile (growing) rabbits during the time course of the immunosuppression experiment. (F) To establish the extent of immunosuppression, T cell counts of the three immunosuppressed rabbits shown in panel D are shown as gray columns (rabbits A, B, and C); T cell counts of a typical immunocompetent animal are shown as black columns.

Following experimental ROPV infection of the lingual mucosa of immunocompetent rabbits, papillomas form within 4 weeks and immune-mediated regression is complete by 8 weeks postinfection, with no evidence of lesion presence by 10 weeks (12). Spontaneous regression of papillomas is a universal outcome of experimental ROPV infection, with the detection of both viral DNA and RNA in the absence of apparent disease for at least a year postinfection (12). The spontaneous reactivation of ROPV to form a productive infection occurs very rarely in immunocompetent animals, suggesting that latent viral genomes are held in check by the host immune system (12). Interestingly, the administration of the immunosuppressive drug cyclosporine to domestic rabbits can prevent the regression of cutaneous CRPV papillomas (15) and delay the regression of ROPV oral papillomas (16). To examine this with a view to developing an immunosuppressive regimen suitable for reactivation studies, seven female New Zealand White rabbits were infected with ROPV prepared from rabbit warts with permanent marking of the infected sites with tattoo ink as previously described (12). Concurrently, 15 mg/kg cyclosporine (Sandimmun; Sandoz Ltd., United Kingdom) was administered by subcutaneous injection three times per week to four of the rabbits. As expected, papillomas had regressed completely at 8 weeks postinfection in the control rabbits and by 9 weeks postinfection in the three cyclosporine-treated rabbits. In our initial studies, therefore, we were able to delay papilloma regression by only 7 days or so and were unable to prevent regression completely. Interestingly, although a slight reduction in CD8^+^ cells was apparent after 8 weeks of cyclosporine treatment compared to controls, there was no significant decrease in CD4^+^ cells, in accordance with the limited effect on the timing of regression that was seen. All of the rabbits showed adverse side effects upon cyclosporine treatment, including inappetence, lethargy, reduced weight gain, and abscess formation at the injection sites, as has previously been reported (17, 18), with one rabbit having to be culled at 5 weeks postinfection because of weight loss and respiratory disease (severe pulmonary abscess formation). Our efforts to optimize the cyclosporine dosing schedule were confounded by severe weight loss problems among the treated animals, which we concluded would limit our capacity to initiate the long-term immunosuppression experiments required for reactivation studies. To overcome these difficulties, we therefore decided to administer a lower level of cyclosporine together with the corticosteroid dexamethasone (which reduces CD4 and CD8 cell counts in rabbits), in order to reduce adverse responses (19). Concurrent with the experimental infection of four rabbits with ROPV, we initiated triweekly administration of cyclosporine at 15 mg/kg and biweekly administration of 0.5 mg/kg dexamethasone (Dexadreson; MSD Animal Health Ltd., United Kingdom) by subcutaneous injection into three animals. In the infected control rabbit and in one immunosuppressed rabbit, all papillomas had completely regressed by 10 weeks postinfection, but in two immunosuppressed rabbits, papillomas were present and continued to grow until drug administration ceased at 15 weeks postinfection (Fig. 1A). Although one rabbit was subsequently culled because of excessive weight loss, the other retained papillomas until week 19 (i.e., 4 weeks after termination of immunosuppression) before regressing. In both animals, papillomas appeared to grow laterally in all directions, eventually coalescing to form large confluent lesions on the tongue. Papillomas also appeared at sites that had not undergone experimental infection, suggesting that immunosuppression predisposed to autoinfection at new sites (Fig. 1B and C). A repeat of this experiment confirmed our results, with papillomas persisting beyond 12 weeks postinfection in all three immunosuppressed rabbits but not in the control (Fig. 1D). The immunosuppressed rabbits maintained their appetite and continued to gain body weight (Fig. 1E) but showed lower peripheral blood T cell counts than those of control animals and those measured prior to immunosuppression (Fig. 1F). In all instances, the size of the papillomas appeared to correlate inversely with the total white blood cell count, with the rabbit with the lowest white cell count developing the largest papillomas.

### Immunosuppression leads to an elevation of the viral DNA copy number at sites of previous disease.

**FIG 2:**
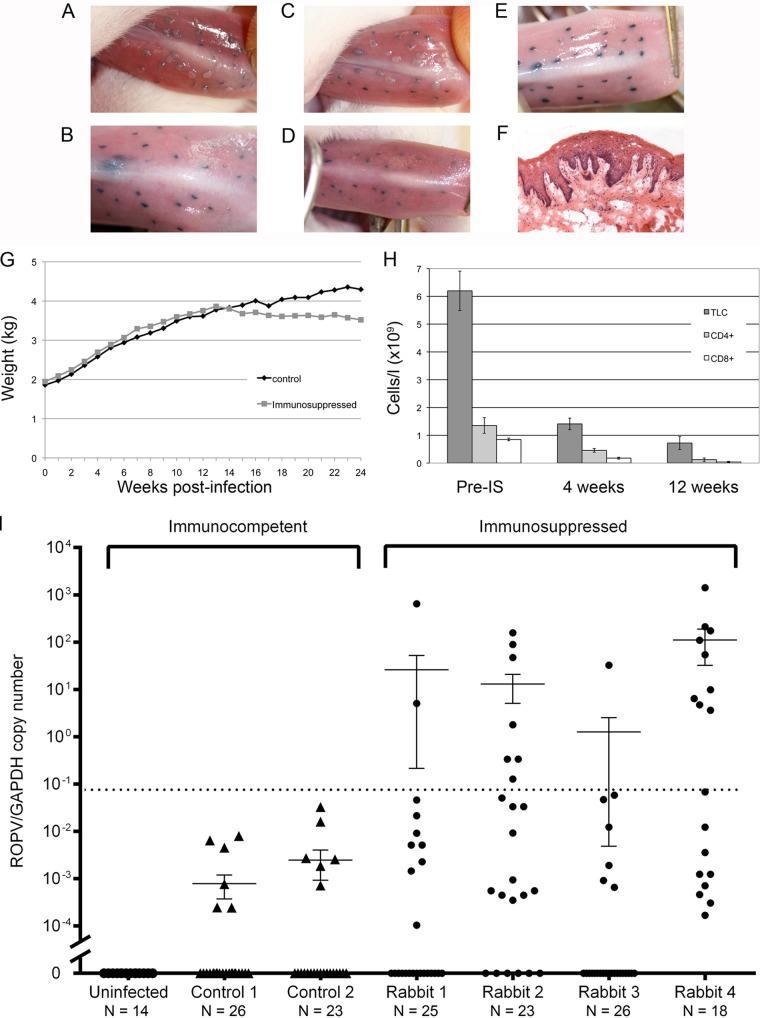
(A to D) Typical appearance of tattoo-marked tongue sites in two ROPV-infected rabbits at 4 weeks postinfection (A and C) and 12 weeks postinfection (B and D). At 4 weeks postinfection (A and C), papillomas are apparent overlying tattoo-marked infection sites. At 12 weeks postinfection in the absence of immunosuppression (B and D), only tattoo marks remain at previous sites of infection. (E) The appearance of tattoo marks in the uninfected control rabbit in which no papilloma lesions developed during the course of the study are indistinguishable from the regressed sites shown in panels B and D. (F) Histology of a single microlesion (with typical papilloma features) detected in rabbit 2 at 12 weeks postimmunosuppression. (G) In adult rabbits, the cyclosporine-dexamethasone immunosuppression regimen generally caused weight loss, with rabbits being culled when their weight loss exceeded 20% of their total body weight. The graph shown is typical of what was seen in repeated experiments. (H) Decline in total lymphocyte counts (TLC; dark gray) and CD4- and CD8-positive (light gray/white) lymphocyte levels following the onset of immunosuppression (IS) in the adult infected rabbits shown in panel I. (I) Numbers of ROPV copies per copy of GAPDH DNA at sites of previous infection in postregression control rabbits (triangles) and postregression immunosuppressed rabbits (circles). Cyclosporine-dexamethasone was administered at week 12 to the immunosuppressed animals, and viral copy numbers were measured at individual tattoo-marked sites at week 24. ROPV/GAPDH copy numbers below the dotted line are those that are typically observed in rabbits with a latent infection or where no ROPV is detectable (12). Elevated copy numbers were seen only at tattoo-marked sites in the immunosuppressed animals.

**FIG 3:**
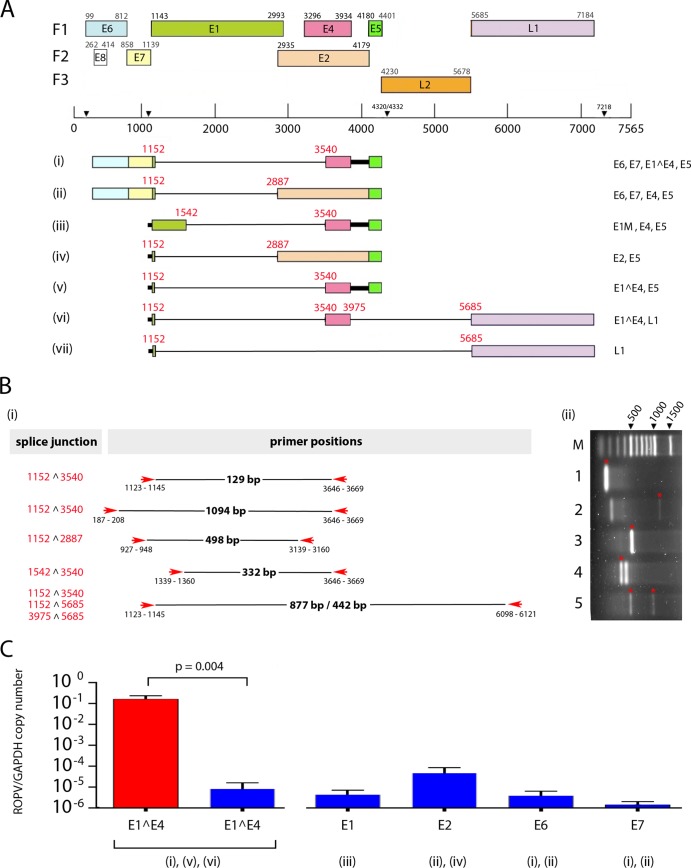
(A) ROPV transcript map based on the analysis of cDNA prepared from a productive rabbit papilloma by a previously described methodology (26). The positions of splice donor and acceptor sites were determined experimentally and are shown in red. Individual transcripts along with their coding capacities are numbered i to vii at the bottom. The positions of the open reading frames and predicted promoter and polyadenylation sites have been reported previously (27) and are marked at the top. (B) Primers used for PCR amplification of ROPV cDNA fragments are indicated by red arrows, and the observed sizes of the amplified products are shown in black. For each primer pair, the amplified bands used for sequence analysis are shown in the gel image to the right and are marked with red asterisks. In addition to specific ROPV sequences, two additional bands were amplified in lanes 2 and 4 as a result of mispriming to cellular sequences. The specific splice junctions present in each PCR fragment are shown on the left. The sequences of the primers used have been reported previously (12). (C) The relative levels of transcripts containing the E1^E4 splice junction (species i, v, and vi) were examined in productive papillomas (red column) and at sites of latent infection (adjacent blue column). The E1^E4 primer pair gave rise to a 129-bp fragment, as shown in track 1 in panel Bii. An approximately 4-log-fold difference in levels was apparent. In latent infections, the relative level of transcripts spanning the E1 (species iii), E2 (species ii and iv), and E6 and E7 regions (species i and ii) are shown to the right of the graph (blue columns) along with the identified transcripts that span these regions.

Having established a schedule for drug-induced immunosuppression that was reasonably well tolerated and prevented papilloma regression in immature (growing) rabbits, we next went on to apply this procedure to mature rabbits that had resolved the disease and had latent infections. Six rabbits were infected with ROPV prepared from rabbit warts, with one rabbit undergoing a mock infection in which the tongue was scarified but without the application of virions. At 4 weeks postinfection, rabbits were examined thoroughly under sedation to confirm that lesions had formed at all of the ROPV-inoculated sites (Fig. 2A and C) but were absent from the mock-infected control (Fig. 2E). When examined again at 8 and 12 weeks postinfection, papillomas were no longer visible in any of the infected rabbits, indicating that immune-mediated spontaneous regression had occurred (Fig. 2B and D). The absence of active infection at 12 weeks postinfection was subsequently confirmed by immunostaining with E4, MCM, and L1 antibodies on tongues taken from culled animals as described previously (12). In keeping with our previous rigorous analysis of >600 regressed tissue slices, we did not find evidence of active infection beyond papilloma regression in immunocompetent animals, either at the level of pathology or by immunofluorescence (12). To examine the effect of immunosuppression postregression, we started four of the rabbits on immunosuppressive treatment at 12 weeks postinfection with triweekly 15 mg/kg cyclosporine and biweekly 0.5 mg/kg dexamethasone and kept two ROPV-infected rabbits as immunocompetent controls. This immunosuppressive regimen was well tolerated (Fig. 2G) and was continued for a further 12 weeks (i.e., until 24 weeks postinfection), after which time all of the rabbits were culled. Examination of the rabbits under sedation at 16, 20, and 24 weeks postinfection did not reveal the presence of visible papillomas in the immunosuppressed animals, the immunocompetent ROPV-infected controls, or the uninfected rabbits. Immunosuppressed animals showed a significant drop in total blood lymphocytes, more specifically, in CD4^+^ and CD8^+^ cell numbers, after 4 and 12 weeks of immunosuppression compared to the preimmunosuppression levels (Fig. 2H). Immunosuppressive treatment was not continued beyond 12 weeks because of a general appetite loss and a decline in body weight beyond 20% of the starting weight. Tattoo-marked tongue infection sites were dissected from culled animals, and ROPV DNA copy numbers were determined relative to the glyceraldehyde 3-phosphate dehydrogenase (GAPDH) copy numbers by real-time PCR (12). From this we established the ROPV genome copy number per cell as previously described. ROPV DNA could not be detected in any of the 13 tattoo-marked mock-infected sites that were examined, even though this rabbit was housed along with ROPV-infected animals in a separate pen in the same room as the rest of the animals. Among the animals that had been infected with ROPV, up to 26 tattoo-marked previous-disease sites were examined per rabbit, with ROPV DNA being found at low levels in 23.1% (control 1 in Fig. 2I) and 26.1% (control 2 in Fig. 2I) of these in the two immunocompetent ROPV-infected control rabbits. These findings are consistent with the formation of a latent infection, with ROPV DNA being detectable at previously infected sites (12). In addition to DNA, ROPV transcripts have also been reported in latent infections (12). Transcript mapping was carried out initially to determine the mRNA species present during productive infection (Fig. 3A and B) and then to establish their levels (relative to GAPDH) postregression. Three mRNA species (i, iv, and vi in Fig. 3A and B) were identified that spanned the major E1^E4 splice junction (nucleotides 11523̂540), with the levels of these ROPV transcripts declining from approximately 1 copy/10 copies of GAPDH mRNA to 1 copy/10,000 copies of GAPDH mRNA during latency (Fig. 3C). Because of the diversity of expression expected as ROPV-infected cells undergo epithelial differentiation, we cannot be certain where particular transcripts are produced but assume (as with other papillomavirus infections) that species v will predominate during productive infections. At latent sites, transcripts capable of expressing E2 (species ii and iv) were present at levels similar to those of transcripts that spanned the E1^E4 splice junction, with those encoding E6 and E7 (species i and ii) being present at a lower abundance. This is reminiscent of what has been reported previously in the CRPV system, where E1 and E2 transcripts may contribute to genome maintenance, with E6 and E7 expression following UV irradiation (14). As we expected, ROPV DNA (and RNA) was not identifiable at all previously infected sites, either because of the low DNA copy number, the imprecision of the tattoo marking system, or possibly also successful disease clearance by the host immune system. In immunosuppressed rabbits, ROPV DNA was present at up to 100% of the previously infected sites, and at sites where ROPV DNA could be detected, the viral genomic copy number was typically 3- to 5-log-fold higher in the immunosuppressed rabbits than in the immunocompetent controls. All four of the immunosuppressed rabbits contained at least one previously infected site that harbored ROPV DNA at levels typically seen only in rabbits with an active infection, despite there being no evidence of grossly visible papillomas. This was not seen in the immunocompetent control animals either here or in our previous study (12). Overall, 19.6% of the tattoo-marked sites taken from the immunosuppressed rabbits showed levels of ROPV DNA that were within the range typically seen during an active infection. Although the limited availability of tissue precluded an analysis of transcription patterns upon immunosuppression, the available formalin-fixed residual tissue permitted the sectioning of tissue from two tattoo-marked infected sites in each of the four immunosuppressed rabbits. At one of the sites examined in rabbit 2, a microscopic papilloma was found above a tattoo mark, suggesting that reactivation may, in some instances, be manifested as microlesions (Fig. 2I and F).

### ROPV persists in tongue tissue grafted onto immunodeficient mice but does not undergo reactivation.

**FIG 4:**
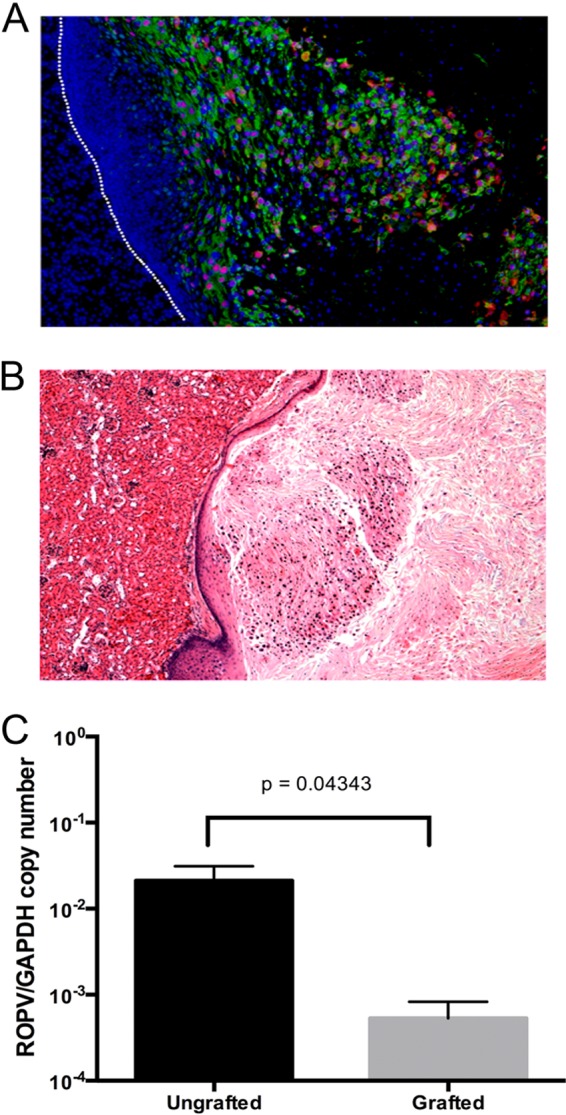
(A) Successful completion of the ROPV life cycle in infected tongue epithelium grafted under the kidney capsules of SCID mice was confirmed by immunostaining for the viral E4 protein (green) and by fluorescence *in situ* hybridization to detect ROPV DNA (red). (B) Analysis of productively infected tissue from SCID mice revealed a characteristic papilloma pathology in grafted epithelium. The images in panels A and B were captured with a 20× objective. (C) The characteristic pathology shown in panel B was absent from latently infected tissue, even at 16 weeks postgrafting. The ROPV copy number per copy of GAPDH was higher before grafting (black column, ungrafted), with the copy number declining by 16 weeks under the kidney capsule (gray column, grafted), which is indicative of latent persistence but not reactivation.

The significant problems with intolerance of rabbits to cyclosporine were insurmountable in our experiments, and we were unable to reliably immunosuppress rabbits for longer than 12 weeks. Despite this difficulty, we went on to repeat the infection and immunosuppression experiments by infecting a further cohort of nine rabbits with ROPV and with induction of immunosuppression at 16 weeks postinfection in seven rabbits for a period of a further 16 weeks. In all infected rabbits, papillomas had formed and regressed as expected by 12 weeks postinfection, and both immunocompetent rabbits survived until the end of the experiment with no adverse effect of signs of papilloma recurrence. We were unable to sustain the immunosuppressed rabbits for the planned duration of the experiment, with all seven developing adverse clinical symptoms as a consequence of cyclosporine and dexamethasone administration (weight loss and severe abscess formation at injection sites and in the oral cavity) that necessitated culling on humane grounds. As a result, we were unable to examine the effects of immunosuppression more extensively in this additional group of animals. As an alternative, therefore, we decided to place infected tissues into an environment devoid of T cell immunity by grafting them under the kidney capsules of immunodeficient mice (20). By incubating rabbit tongue tissue with ROPV virions and then grafting it into mice with severe combined immunodeficiency (SCID mice), we confirmed that the environment was supportive of graft growth and survival, as well as allowing completion of the productive ROPV life cycle (Fig. 4). Tissues were then taken from the tongues of previously infected rabbits culled at 12 to 16 weeks postinfection. Quantification of ROPV DNA in 34 tissue samples showed evidence of ROPV persistence at 19 sites. Another 24 tissue samples were grafted into SCID mice for 16 weeks before being recovered for quantification of ROPV DNA, and in all 24 samples, low levels of ROPV DNA were found. Levels of ROPV DNA were lower than in ungrafted control tissues, which was consistent with the persistence of a latent infection but not with reactivation (Fig. 4C). The unexpected loss of ROPV genomes in the graft system may reflect the different growth pressures on latently infected cells (e.g., stem cells) in this system. Although we anticipate that this immunodeficient environment should be permissive for latent reactivation, we suspect that additional factors are necessary for this to occur that are not provided in the kidney capsule system. Such factors include mechanical irritation and stimulation of a “wound-healing” response, which can influence the papillomavirus copy number in other model systems and which can occur in an environment susceptible to repeated irritation such as the tongue.

On the basis of our previous studies, we proposed a model of papillomavirus latency in which viral gene expression is held in check in the epithelial basal layer by constant immunosurveillance and in which immunosuppression may be permissive of increased virus activity, as suggested in humans (12, 21, 22). Our current data support this hypothesis and show for the first time that suppression of the adaptive immune system can lead directly to an elevation of the viral DNA copy number at sites of previous infection, which is consistent with reactivation from latency. In our experiments, however, it appears that additional reactivation studies with rabbits will be difficult because of the problems inherent in the long-term administration of systemic immunosuppressive drugs to rabbits. A papillomavirus of the laboratory mouse Mus musculus has recently been described, and although MmuPV1 is likely to have distinct evolutionary adaptations that reflect its position as a Pi papillomavirus, it is anticipated that this model will be useful in further enhancing our understanding of latent infections (23–25).
